# Vitamin C Deficiency as a Risk Factor for Bell’s Palsy: A New Association

**DOI:** 10.7759/cureus.21143

**Published:** 2022-01-12

**Authors:** Raktim Swarnakar, Shiv L Yadav

**Affiliations:** 1 Physical Medicine and Rehabilitation, All India Institute of Medical Sciences, New Delhi, New Delhi, IND

**Keywords:** immunological, gingival hypertrophy, neuro-rehabilitation, bell`s palsy, vitamin - c

## Abstract

A 28-year-old female developed gum hypertrophy after five months of Bell’s palsy (BP). The vitamin C level was severely low. After vitamin C supplementation for one month, gingival hypertrophy was completely resolved. Facial deviation also improved following rehabilitation. Vitamin C is commonly considered as an antioxidant, anti-inflammatory, and immunomodulator, and it hastens recovery of neuritis caused by herpes (cause of BP). BP too has an immune-inflammatory background. To the best of our knowledge, for the first time, vitamin C deficiency has been reported as a cause or triggering/risk factor for Bell’s palsy and at the same time immune-inflammation triggered in BP also may lead to vitamin C deficiency as existing vitamin C in the body starts scavenging free radicals to prevent oxidative damage. Vitamin C levels must be checked in all cases of BP, and intake of vitamin C-rich food should be encouraged in people who are at risk of developing BP.

## Introduction

Bell’s palsy (idiopathic facial paralysis) is an acute or subacute weakness of the facial muscles innervated by the seventh cranial nerve (facial nerve). The exact pathogenesis of Bell’s palsy is not known, but viral infections and immunoinflammation are postulated as important causes of Bell’s palsy [1]. On the other hand, vitamin C is commonly considered as an antioxidant and anti-inflammatory and vitamin C also hastens recovery in some cases of neuritis caused by herpes simplex viruses (one of the causes of Bell’s palsy). Low vitamin C impairs immunity as well. In this context, we wish to highlight the vitamin C deficiency as a cause or triggering factor or risk factor for Bell’s palsy and at the same time immune-inflammation triggered in Bell’s palsy also leads to vitamin C deficiency since existing vitamin C in the body starts scavenging reactive oxygen species to prevent oxidative damage (i.e., neuritis). To the best of our knowledge, this is the first case report where this new association and cause-and-effect relationship of vitamin C and Bell’s palsy has been reported.

## Case presentation

**Figure 1:**
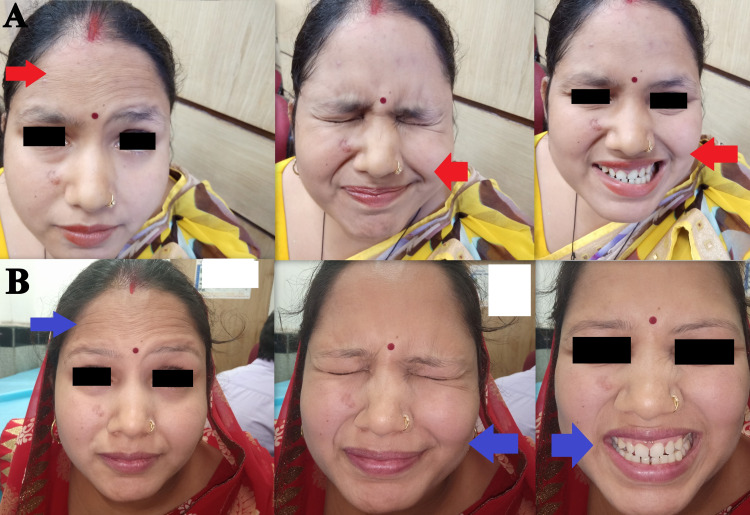
Bell’s palsy A) Facial deviation before rehabilitation (red arrow indicates loss of forehead wrinkles on right side and facial deviations); B) Improvement in facial deviation after rehabilitation (blue arrow indicates improvement in forehead wrinkles and facial deviations).

**Figure 2:**
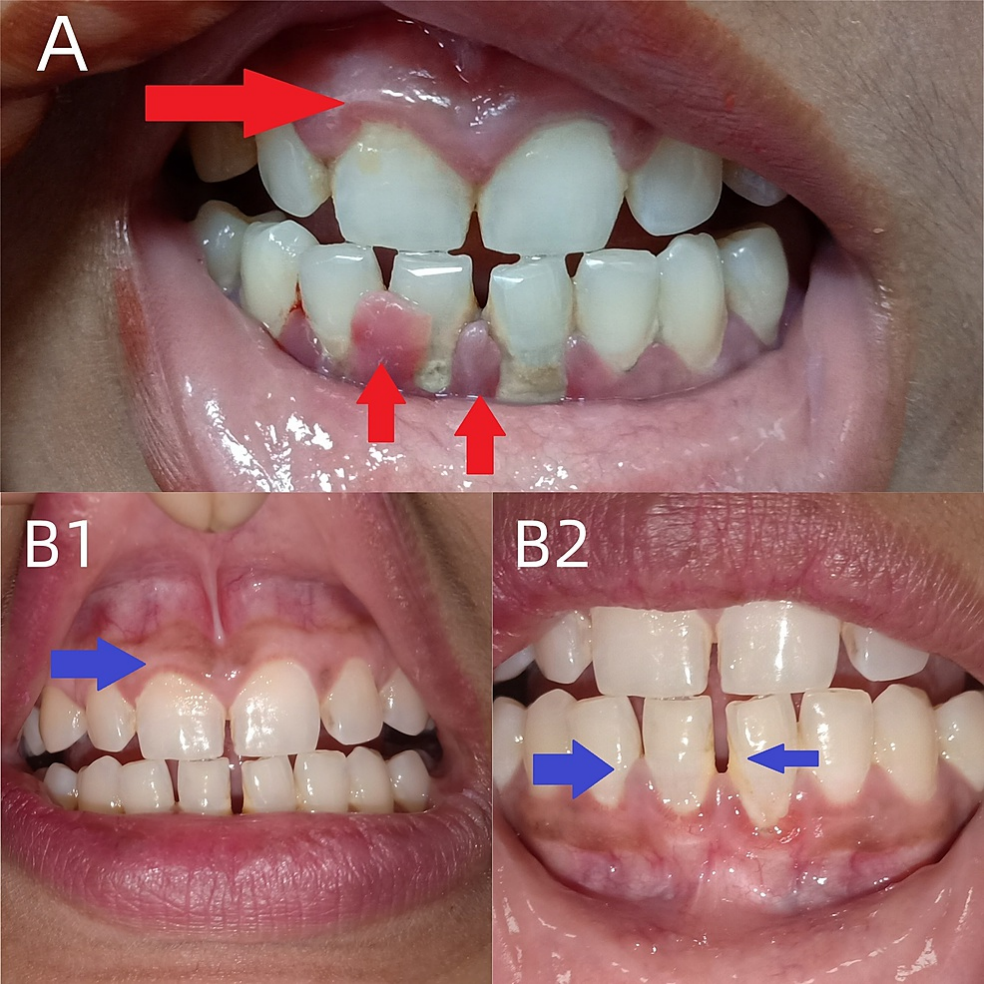
Gingival hypertrophy and its resolution after vitamin C therapy A) Gingival hypertrophy (upper and lower gingiva, red arrows indicate hypertrophy); B1) resolution (blue arrow indicates resolution) of upper gingival hypertrophy; B2) resolution (blue arrows) of lower gingival hypertrophy.

A 28-year-old housewife presented to rehabilitation outpatient setting with right-sided lower motor neuron type seventh cranial nerve palsy (Bell’s palsy) of three and half months duration. She noticed facial deviation following a one-day mild headache. She had a history of intake of prednisolone 20 mg/day for 10 days along with multivitamins (vitamin B complex and vitamin C) after consulting the doctor. In the rehabilitation medicine setting, routine rehabilitation and physiotherapy with facial exercises were followed and improvement of facial deviation was observed (Figure 1, panels A and B). After three weeks of rehabilitation setting visit, one day she observed gum hypertrophy and she reported to our outpatient setting. She also reported bleeding from gum during brushing on a few occasions. On clinical examination, gingival hypertrophy was noted (Figure 2, panel A), and it was diffuse in the upper and lower gingiva. The patient had no other health comorbidities; there was no history of fever during or after facial deviation, and also she had no fever at the time of gum hypertrophy. There was no history of joint pain (except mild fatigue) and bowel bladder issues. She denied any history of smoking and alcohol intake. She had no history of intake of any other medications. The patient also had no previous history of tuberculosis or contact with tuberculosis and had no family history of similar illness or malignancy.

She was advised routine blood investigations like complete hemogram, erythrocyte sedimentation rate (ESR), fasting and postprandial blood sugar, liver function tests (LFTs), kidney function tests (KFTs), and also serum vitamin C level. Routine blood investigations were within normal limits. But vitamin C level was found to be severely low, reported as <0.1 mg/dl (0.3-2.7 mg/dl). Routine investigations were done to rule out the underlying dysfunction of other systems. Tablet vitamin C (500 mg) once daily along with vitamin C-rich foods was advised. She was also advised facial physiotherapy for facial deviation.

Following rehabilitation and physiotherapy, the facial deviation was improved (Figure 1, panels A and B). Gingival hypertrophy completely resolved after one month of vitamin C intake (Figure 2, panels A, B1, and B2). At six-month follow-up, no recurrence of gingival hypertrophy was observed. 

## Discussion

To the best of our knowledge, for the first time, such association has been reported. Putting into broader perspectives, we have discussed all possible scenarios. First, vitamin C deficiency might have triggered Bell’s palsy, and second, Bell’s palsy induced low vitamin C level. On the other hand, less possibilities were prednisolone-induced vitamin C deficiency; third, vitamin C deficiency may be an incidental finding. Here, we have discussed those scenarios in the background of available scientific literature.

Vitamin C is considered an antioxidant and free radical scavenger in our body. Thus, it prevents oxidative stress-induced damage in the body. On the other hand, oxidative stress is also a known triggering factor for Bell’s palsy. Furthermore, viral infections and immune-inflammation are among the postulated causes of Bell’s palsy, but exact pathogenesis is still not known. Studies showed that immunosuppression and viral reactivation following stress can cause Bell’s palsy [2,3]. Interferons that combat viral infections are also found to be increased in Bell’s palsy [4]. Vitamin C improves immunity, and its deficiency showed impaired immunity in studies. Vitamin C is also found to modulate cytokines and interferon production [5]. Vitamin C is also known to help in neural regeneration [6,7] and has neuroprotective roles [8]. Considering those facts, vitamin C deficiency may trigger Bell’s palsy in susceptible individuals.

Furthermore, increased oxidative stress is found in Bell’s palsy, and increased oxidative stress is also known to cause depletion of vitamin C since existing vitamin C in the body actively participates in scavenging free radicles or reactive oxygen species to prevent oxidative damage (i.e., neuritis). This explains how Bell’s palsy may trigger vitamin C deficiency.

In our case, we observed the gingival hypertrophy and low serum vitamin C level in the middle of the treatment and especially after the onset of facial deviation. Her gingival hypertrophy was not present before the onset of facial deviation; hence serum level was not done during that time; this fact does not exclude the possibility of low vitamin C level before facial deviation. The reason is that symptomatic manifestation of vitamin C deficiency occurs after 8 to 12 weeks of inadequate intake. Hence even if she had a deficiency, she did not develop clinically evident gum hypertrophy. Generally, less than 0.2 mg/dl is consistent with scurvy, but in our case, such a low level did not stay for long duration to cause bony pathology.

Prednisolone-induced scurvy was previously reported in a patient with rheumatoid arthritis [9], but for such a short duration and in low dose, the steroid can rarely cause any side effects, and that too scurvy. Looking at the timeline of our case and history of intake of multivitamins (vitamin B complex and vitamin C) during steroid intake excludes this possibility of steroid-induced vitamin C deficiency.

**Figure 3:**
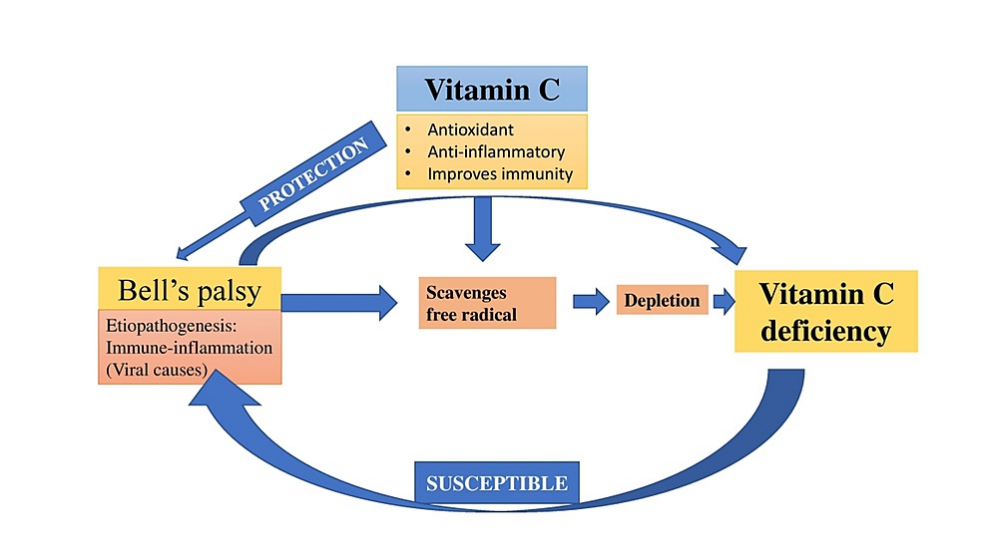
Vitamin C and Bell’s palsy Schematic illustration showing association of vitamin C and Bell’s palsy.

Coming to the last possibility of incidental finding of vitamin C deficiency, it is a fact that vitamin C deficiency may be common, but symptomatic vitamin C deficiency is very rare nowadays. Furthermore, her history of diet pattern, the timeline of symptoms, no bony involvement, and no recurrence of gingival hypertrophy all indicate that the reason for onset vitamin C deficiency was within the timeline of Bell’s palsy. No bony involvement also strongly indicates that this deficiency was not for a long duration. This new association of vitamin C deficiency and Bell’s palsy and its interrelationship and vicious cycle has been shown in the schematic illustration (Figure 3).

## Conclusions

Our case signifies that vitamin C deficiency is a risk factor/triggering factor of Bell’s palsy. On the other hand, Bell’s palsy also may induce vitamin C deficiency. Serum vitamin C levels should be checked in every patient with Bell’s palsy. Intake of vitamin C-rich foods should be encouraged in the general population especially in those who already possess risk factors for Bell’s palsy and in patients with Bell’s palsy as well. Supplementation with vitamin C tablets should only be started if deficiency is found in serum levels.
